# Single-Step Electrodeposition of ZnO Nanoparticles Decorated (111)-Textured Cu_2_O Films with Enhanced Photoelectrochemical Properties

**DOI:** 10.1021/acs.inorgchem.5c02573

**Published:** 2025-08-12

**Authors:** Yu-Hao Huang, Yung-Tang Chuang, Hao-Wu Lin, Chien-Neng Liao

**Affiliations:** † College of Semiconductor Research, 34881 National Tsing Hua University, Hsinchu 30013, Taiwan; ‡ Department of Materials Science and Engineering, National Tsing Hua University, Hsinchu 30013, Taiwan

## Abstract

Cuprous oxide (Cu_2_O) is a promising material for photoelectrochemical water splitting due to its favorable band structure, environmental acceptability, nontoxicity, and ease of fabrication. In this study, (111)-textured Cu_2_O films decorated with numerous ZnO nanoparticles (NPs), namely ZCO films, are synthesized by a facile one-step electrodeposition method. The mechanism for forming ZnO NPs during the electrodeposition of Cu_2_O films is proposed. Based on the Mott–Schottky analysis and electrochemical impedance spectroscopy measurements, the presence of ZnO NPs on the Cu_2_O films enhances band bending and reduces charge transfer resistance to the electrolyte, which is advantageous for separating photoexcited electron and hole carriers in the illuminated ZCO films. Additionally, excess oxygen vacancy defects may facilitate the interdefect hopping of photoelectrons by minimizing the recombination probability of transporting photocarriers within the ZCO films according to photoluminescence spectroscopy analysis. The ZCO film exhibits a 40% increase in photocurrent density over the pristine Cu_2_O film in 1 M Na_2_SO_4_ solution under AM1.5G illumination. This study provides a facile synthesis route for creating high-performance Cu_2_O-based photocathodes through heterojunction and crystal defect engineering.

## Graphical abstract

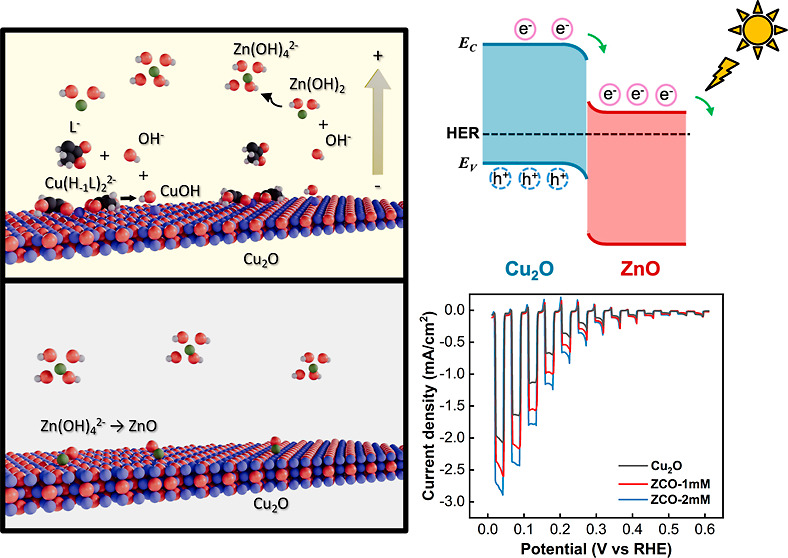

## 1 Introduction

Solar power is a clean and renewable energy source; however, its intermittent availability due to the day–night cycle poses challenges to ensuring a consistent energy supply.^1^ One feasible solution is to convert solar energy into storable chemical fuels such as hydrogen gas through photoelectrochemical (PEC) water splitting. Hydrogen has a high energy density and a heating value of 141.9 MJ kg^–1^, making it an excellent fuel for long-term energy storage and usage.^2,3^ The PEC process typically involves three critical steps.^4^ First, photoactive semiconductors absorb sunlight to generate electron–hole (*e*–*h*) pairs. A low *e*–*h* generation rate occurs when photoactive semiconductors respond only to a limited portion of solar light spectrum. For example, TiO_2_ has a wide bandgap of 3.2 eV and can be activated by ultraviolet (UV) light with wavelengths below 388 nm only, leaving a significant portion of the visible to near-infrared light spectrum largely unutilized. Techniques such as band engineering and impurity doping can enhance light absorption and broaden the operational spectrum of photoactive semiconductors.^5^ Second, the driving force for photocarriers moving toward the electrode/electrolyte interface depends on the energy band positions, i.e. conduction band minimum (CBM) and valence band maximum (VBM) relative to the potential of redox couples (e.g., H^+^/H_2_ and H_2_O/O_2_) in the electrolyte. In addition, recombination or trapping of photoelectrons within the photoactive semiconductor may result in a decrease in PEC efficiency.^6^ Third, photoelectrons transporting to the electrode/electrolyte interface will be consumed during the hydrogen evolution reaction (HER). This step is often constrained by slow reaction kinetics and catalytic activity of photoactive semiconductors. Modifying the photoelectrodes with cocatalysts is a common strategy to accelerate HER kinetics.^7^

Among various photoactive semiconductors, cuprous oxide (Cu_2_O) has emerged as a promising material for PEC water-splitting applications owing to its favorable bandgap (2.0–2.5 eV), which allows it to absorb a significant portion of solar energy in the visible light spectrum. Cu_2_O is also abundant and cost-effective, making it a promising photoactive material for large-scale solar energy conversion applications.^8,9^ However, Cu_2_O is highly susceptible to photodegradation in aqueous environments. This degradation will lower the photon-to-electron conversion efficiency and impact the photoelectrode longevity in PEC systems.^10^ Additionally, low separation efficiency and high recombination rate of photocarriers are also major technical challenges for developing high-performance Cu_2_O-based photocathodes.^11^ Various strategies, including semiconductor sensitization,^12^ p–n junction,^13,14^ Z-scheme heterojunction,^15^ surface passivation,^16–18^ facet control,^19^ and cocatalysts,^20,21^ have been proposed to tackle the above-mentioned problems associated with Cu_2_O photocathodes. Among these techniques, ZnO-modified Cu_2_O has demonstrated potential in solar cells, CO_2_ reduction, and water splitting applications.^22–25^ Note that ZnO has a wide bandgap of 3.37 eV, which can extend light absorption into the UV spectral range.^26^ Cu_2_O exhibits a CBM at −1.3 V vs RHE and a VBM at +0.6 V vs RHE,^9,27^ whereas the CBM and VBM of ZnO position at −0.5 V and +2.7 V, respectively.^28^ This band position offset (Δ*E*_CB_ ≈ 0.8 eV; Δ*E*_VB_ ≈ 2.1 eV) produces a staggered type-II heterojunction between p-type Cu_2_O and n-type ZnO. The heterojunction creates a built-in electric field at the interface, facilitating the flow of photoelectrons from Cu_2_O to ZnO.^29^ The introduction of Cu_2_O/ZnO heterojunction can be achieved using atomic layer deposition (ALD) or other multistep processes like physical vapor deposition,^30^ hydrothermal techniques,^31^ and electrodeposition.^25^ These methods involve the preparation of Cu_2_O and deposition of ZnO in sequence, making them more expensive and time-consuming. In this study, we developed a facile one-step electrodeposition method to create textured Cu_2_O films decorated with ZnO nanoparticles (NPs) on their surface. We propose a tentative mechanism for the formation of ZnO NPs during the electrodeposition of Cu_2_O. Additionally, we investigate the role of ZnO NPs in improving the PEC efficacy of Cu_2_O photocathodes. The findings will contribute to the design of cost-effective and high-performance Cu_2_O-based photoelectrodes for solar-driven water splitting applications.

## 2 Experimental Section

### 2.1 Sample Preparation

A fluorine-doped tin oxide (FTO) substrate (2 cm × 1 cm) was sequentially cleaned with acetone, isopropanol, ethanol, and deionized water to ensure a contaminant-free surface. The cleaned substrate was then loaded into an electron-beam deposition system with the chamber pumped to a base pressure of 5 × 10^–6^ Torr. Subsequently, a 20 nm-thick Au layer was e-beam evaporated on the FTO at a deposition rate of 0.2 Å/s to improve the electrical conductivity and adhesion of the electrodeposited Cu_2_O layer. The electrolyte for Cu_2_O electrodeposition was prepared by mixing 45 g of CuSO_4_·5H_2_O and 75 mL of lactic acid, followed by the addition of 5 M NaOH to a final volume of 300 mL. The mixture was stirred continuously for 24 h to obtain the Cu­(II)-lactate electrolyte.^32^ The electrolyte of 50 mL in volume was extracted and heated to 58 °C in a temperature-controllable water bath. The pH value of the Cu­(II)-lactate alkaline electrolyte was adjusted to 10.1 by incrementally adding NaOH solution. Additionally, the Cu­(II)-lactate alkaline electrolyte was modified by adding ZnSO_4_ (1 mM and 2 mM) to deposit the ZnO NP-decorated Cu_2_O films, designated as ZCO-1 mM and ZCO-2 mM, respectively. The pristine Cu_2_O and the ZCO films were respectively electrodeposited in a standard three-electrode cell under a constant current density of 6 mA/cm^2^ for 83 s using the electrochemical workstation (6273E, CH Instruments).^27^ The FTO/Au substrate served as the working electrode, while a platinum plate and a saturated calomel electrode were used as the counter and reference electrodes, respectively.

### 2.2 Characterization Methods

The synthesized Cu_2_O-based photoelectrodes were examined by an X-ray diffractometer (XRD, D8 ADVANCE, Bruker) with a grazing angle of 0.5° and a scan range of 20–80°. Their surface morphologies were revealed by scanning electron microscopy (SEM, SU8010, Hitachi). A transmission electron microscopy (TEM, JEM-ARM200FTH, JEOL) was employed to characterize the nanostructure of ZnO NPs on the Cu_2_O film. The elemental composition and valence states of the Cu_2_O and ZCO films were analyzed by using an X-ray photoelectron spectroscopy (XPS, PHI 5000 VersaProbe II, ULVAC-PHI). The elemental composition of Zn and Cu in the pristine Cu_2_O, ZCO-1 mM, and ZCO-2 mM samples were also analyzed by the inductively coupled plasma mass spectrometry (ICP–MS, iCAP TQ, Thermo Fisher Scientific). The optical adsorption characteristics of the prepared electrodes were measured using UV–visible spectroscopy (UV–vis, U-3900, HITACHI) over a wavelength range of 300–800 nm, while the optical emission properties were studied with photoluminescence (PL, iHR550, HORIBA) spectroscopy using a 532 nm excitation laser and an emission detection range of 400–750 nm. A time-resolved photoluminescence (TPRL) analysis was performed using a 375 nm pulsed laser (PicoQuant) with a repetition rate of 2 MHz. Excited photons were collected by a high-sensitivity photomultiplier tube (PMC-100-1, Becker & Hickl GmbH), and photon counts were accumulated with a time-correlated single-photon counting module (SPC-150, Becker & Hickl GmbH), providing detailed insights into photocarrier lifetimes. An electron paramagnetic resonance (EPR, ELEXSYS E-580, BRUKER) spectrometer was employed in analyzing oxygen vacancies in the synthesized Cu_2_O films.

### 2.3 Photoelectrochemical Measurements

Photoelectrochemical measurements were performed using a custom-built electrochemical cell containing a 1 M Na_2_SO_4_ solution (pH = 7). The three-electrode cell, which includes the Cu_2_O-based photocathode, an Ag/AgCl reference electrode (saturated KCl), and a Pt counter electrode, was connected to an electrochemical workstation (6273E, CH Instruments). The photocathode was illuminated from the front side by an AM1.5G solar simulator (LS150, ABET Technology) with an intensity of 100 mW/cm^2^. The photocurrent data were obtained from linear sweep voltammetry (LSV) measurements conducted under chopped illumination within a potential range of 0 to 0.6 V vs Ag/AgCl. The carrier concentration and flatband potential (*E*_fb_) were determined from the Mott–Schottky measurements conducted at a fixed frequency of 1000 Hz within a potential range of −0.2 to 0.2 V vs Ag/AgCl.^33^ An electrochemical impedance spectroscopy (EIS) analysis was employed to evaluate the carrier transport resistance and interfacial properties between the photocathode and electrolyte. The EIS measurement was conducted with a frequency range of 0.1 Hz to 1 MHz under both dark and light conditions.^34^

Safety Statement Standard laboratory safety procedures (lab coats, nitrile gloves, and safety goggles) were followed throughout. No uncommon hazards are noted.

## 3 Results and Discussion

### 3.1 Microstructure and Phase Characterization of ZCO Films

Controlling crystal growth direction is essential for making high-performance photoelectrodes because both charge transport and surface redox reaction depend on the crystal orientations of photoactive materials. Figure 1a-d shows the XRD patterns and SEM micrographs of the pristine Cu_2_O, ZCO-1 mM, and ZCO-2 mM samples, respectively. All three samples exhibit preferential growth along the Cu_2_O <111> direction and tetrahedron-shaped Cu_2_O crystallites on the film surface. The exposed facets of tetrahedron crystallites are Cu_2_O {100} surfaces. Since the <111> direction is the most conductive path in Cu_2_O, the <111> textured Cu_2_O film ensures minimal charge transport resistance within the photocathode.^35^ Moreover, the conduction and valence band edges near the facet surfaces are expected to bend down when the *p*-type Cu_2_O is in contact with the aqueous electrolyte. This bent band structure facilitates photoelectrons to flow from Cu_2_O to the electrolyte. Additionally, Cu_2_O {100} facets are also efficient electron doorways to the electrolyte because their band structures bend more significantly than Cu_2_O {111} facets.^35,36^ Thus, the synthesized Cu_2_O-based films have the optimal crystallographic orientation for use as photocathodes in a PEC water-splitting system.

**Figure 1:**
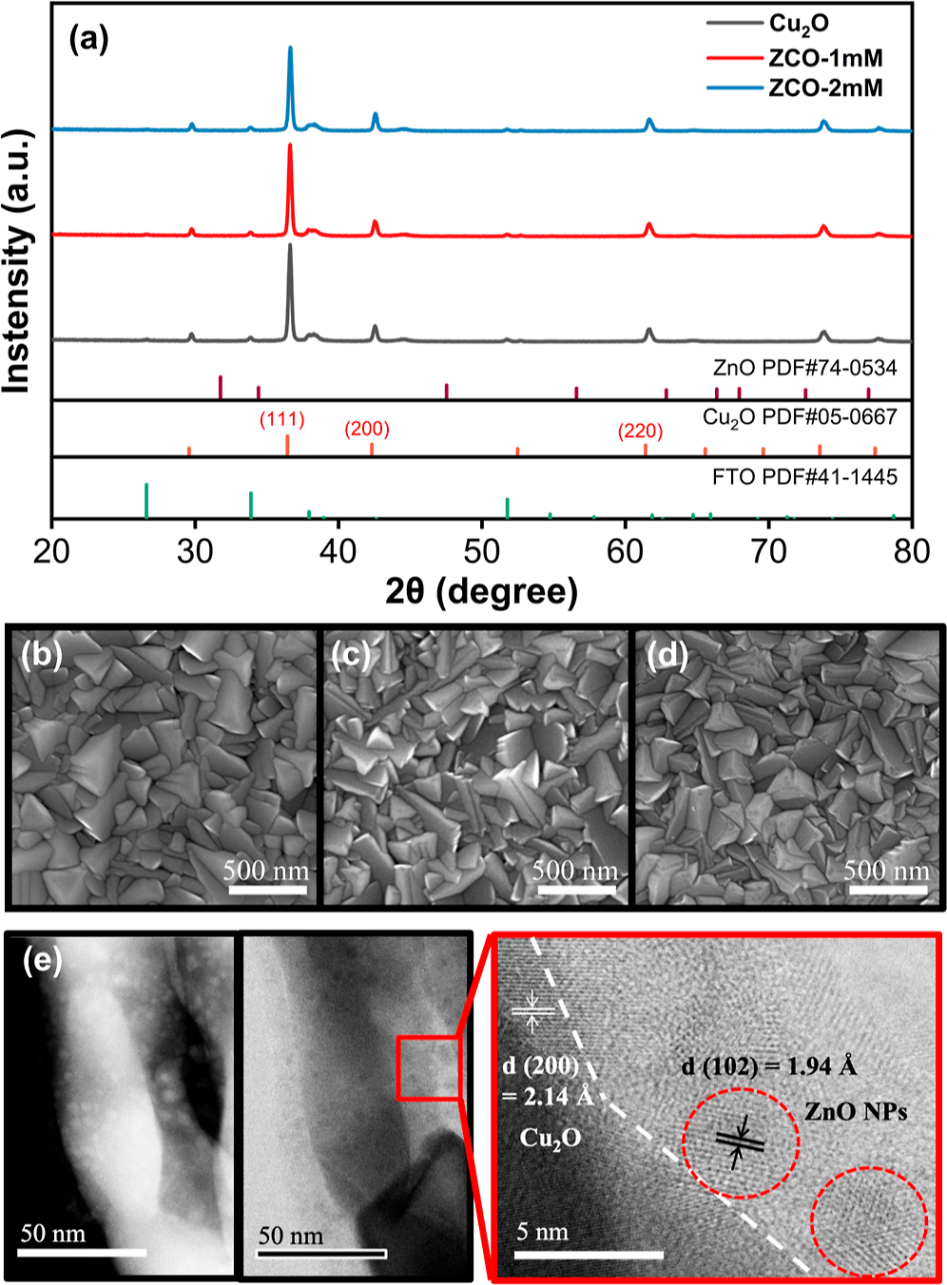
(a) The XRD patterns of the synthesized Cu_2_O and ZCO samples. (b–d) The SEM micrographs of (b) pristine Cu_2_O, (c) ZCO-1 mM, and (d) ZCO-2 mM samples. (e) The dark-field (left) and bright-field TEM images (right) of the ZCO-2 mM sample.

It is worth noting that no ZnO reflections are observed in the XRD pattern of ZCO-2 mM even in a logarithmic scale plot (Figure S1). The SEM micrographs of the ZCO-1 mM and ZCO-2 mM samples could not identify the presence of ZnO NPs either (Figure 1c,d). Thus, we further examined the ZCO-2 mM sample by using high-resolution TEM (HRTEM). Many small NPs (∼5 nm) were observed around the Cu_2_O grains according to the HRTEM images (Figure 1e). In the amplified HRTEM image, the NPs show an interplanar spacing of 1.94 Å, which is consistent with the *d*_(102)_ of the ZnO crystal.^37^ The presence of ZnO NPs in the synthesized ZCO film is thus confirmed.

The distribution of Zn elements in the ZCO films was further investigated by XPS analysis. Figure 2a–c displays the Zn 2p XPS spectra of the pristine Cu_2_O, ZCO-1 mM, and ZCO-2 mM samples. The binding energies of the Zn 2p_3/2_ and Zn 2p_1/2_ core levels are 1021.0 and 1044.2 eV, respectively.^26^ The ZCO-2 mM appears to have more Zn content than the ZCO-1 mM according to the Zn 2p spectral intensity. Additionally, an XPS depth profiling analysis was conducted to reveal the Zn distribution inside the ZCO-2 mM sample (Figure 2d–f). Surprisingly, the Zn 2p spectral intensity declines sharply with increasing depth within the ZCO film. This indicates that ZnO NPs are primarily located on the surface of the ZCO film. Since the ZCO films were electrodeposited in the Zn^2+^ ion-contained electrolyte, Zn elements might be incorporated into the Cu_2_O crystal lattice. We have checked the Cu 2p and O 1s XPS spectra of the Cu_2_O and ZCO samples as well. No significant changes in the binding energies of Cu 2p_3/2_ and Cu 2p_1/2_ core levels were observed (Figure 3a–c). Additionally, the O 1s XPS spectra were deconvoluted into three components, attributed to contributions from lattice oxygen (O_L_), oxygen-related defects (O_D_), and adsorbed oxygen (O_A_) (Figure 3d–f). The O 1s signal at ∼531 eV (O_D_) is possibly associated with −OH or other oxygen-related defects rather than oxygen vacancies. An XPS depth profile of the O 1s spectra indicates that the O_A_ and O_D_ species appear to reside on the surface only because their signals vanish after a brief Ar^+^ bombardment (Figure S2). A quantitative assessment of oxygen vacancies in the Cu_2_O-based films cannot be solely achieved through XPS analysis.^38^ A discussion on the oxygen vacancy defects in the Cu_2_O and ZCO films will be presented later. However, we can conclude that small ZnO NPs tend to form across the surface of the ZCO films based on the TEM and XPS analyses.

**Figure 2:**
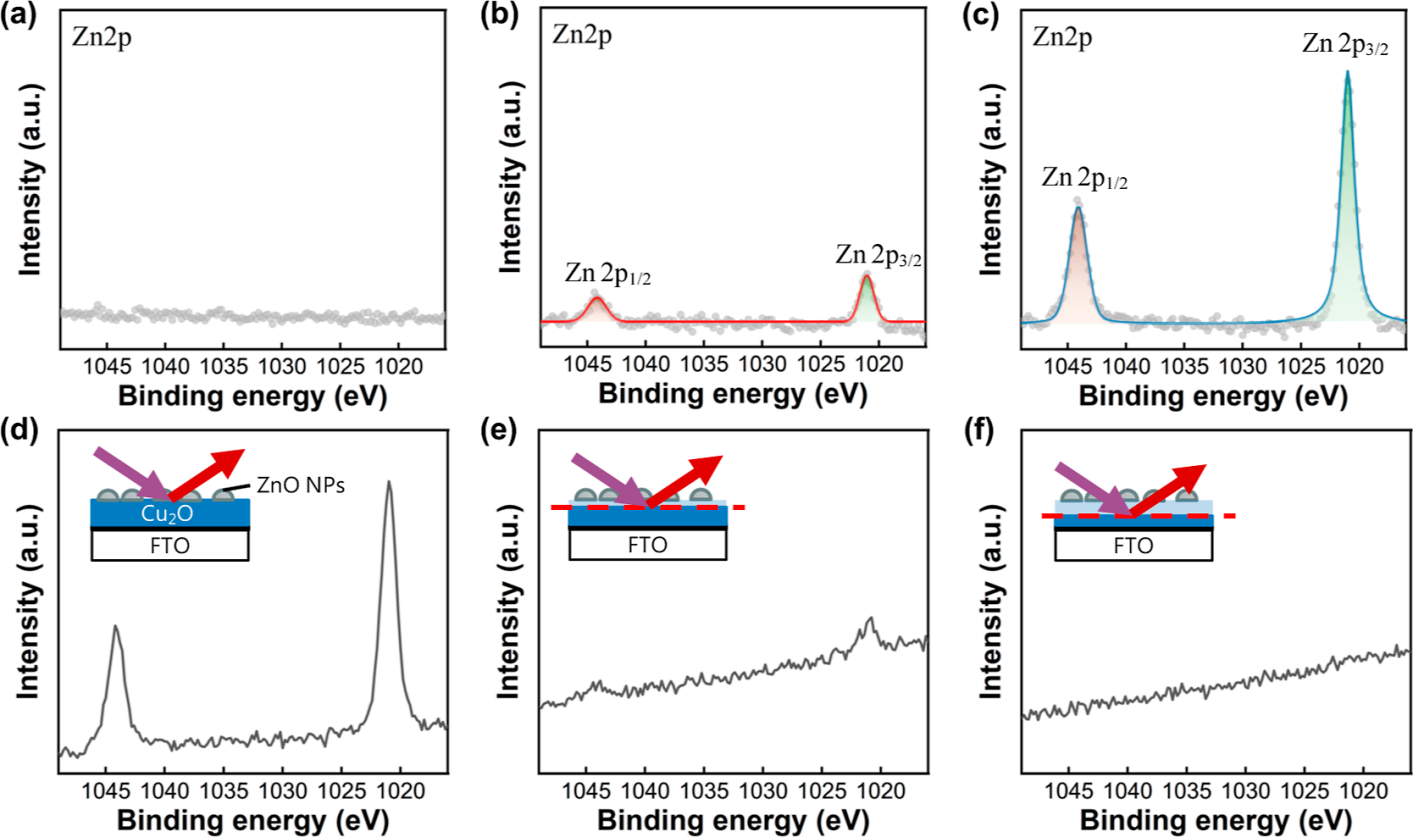
Zn 2p XPS spectra of (a) pristine Cu_2_O, (b) ZCO-1 mM, and (c) ZCO-2 mM samples. An XPS depth profiling analysis of the ZCO-2 mM sample subjected to Ar ion milling for (d) 0 min, (e) 3 min, and (f) 5 min.

**Figure 3:**
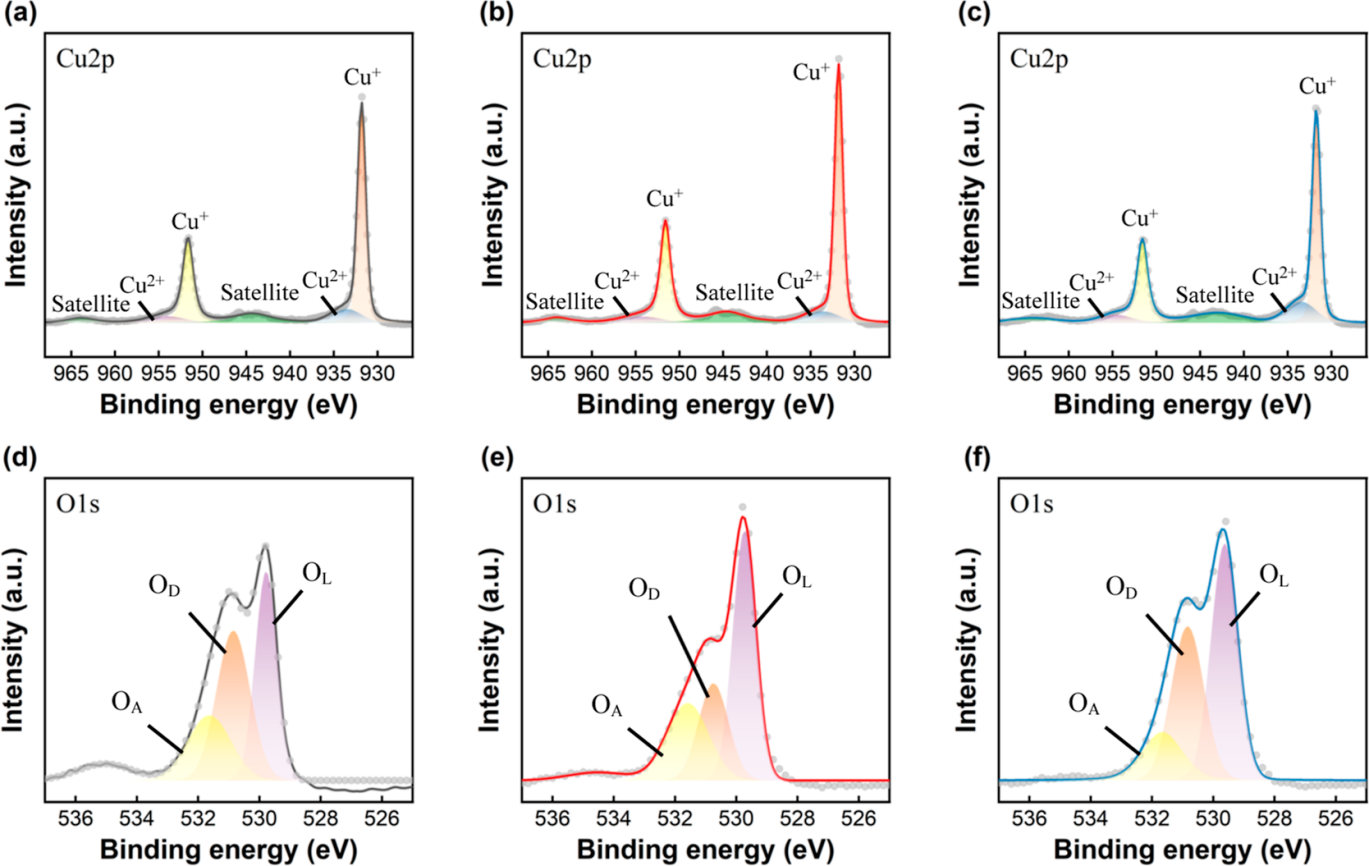
Cu 2p and O 1s XPS spectra of the (a,d) pristine Cu_2_O, (b,e) ZCO-1 mM, and (c,f) ZCO-2 mM samples.

The Cu_2_O-based thin films were electrodeposited using the CuSO_4_ electrolyte containing lactic acid. Cupric ions (Cu^2+^) react readily with lactate molecules by forming various Cu­(II)-lactate complexes, such as Cu­(H_–1_L)­L^–1^ and $Cu{\left(H_{-1}L\right)}_{2}^{2-}$ , in the solution. The L^–1^ and H_–1_L stand for CH_3_CH­(OH)­COO^–^ and CH_3_CH­(O^–^)­COO^–^, respectively.^39^ These Cu­(II)-lactate complexes facilitate the growth of <111>-textured Cu_2_O film in alkaline electrolytes, following a two-step process (Figure 4a): (1) conversion of Cu­(II)-lactate complexes into CuOH through a charge transfer process; (2) dehydration of metastable CuOH into Cu_2_O, as described below^40^

1 $$Cu{\left(H_{-1}L\right)}_{2}^{2-}+2H_{2}O+e^{-}\to CuOH+2L^{-}+OH^{-}$$

2 $$2CuOH\to {Cu_{2}O}_{\left(s\right)}+H_{2}O$$

**Figure 4:**
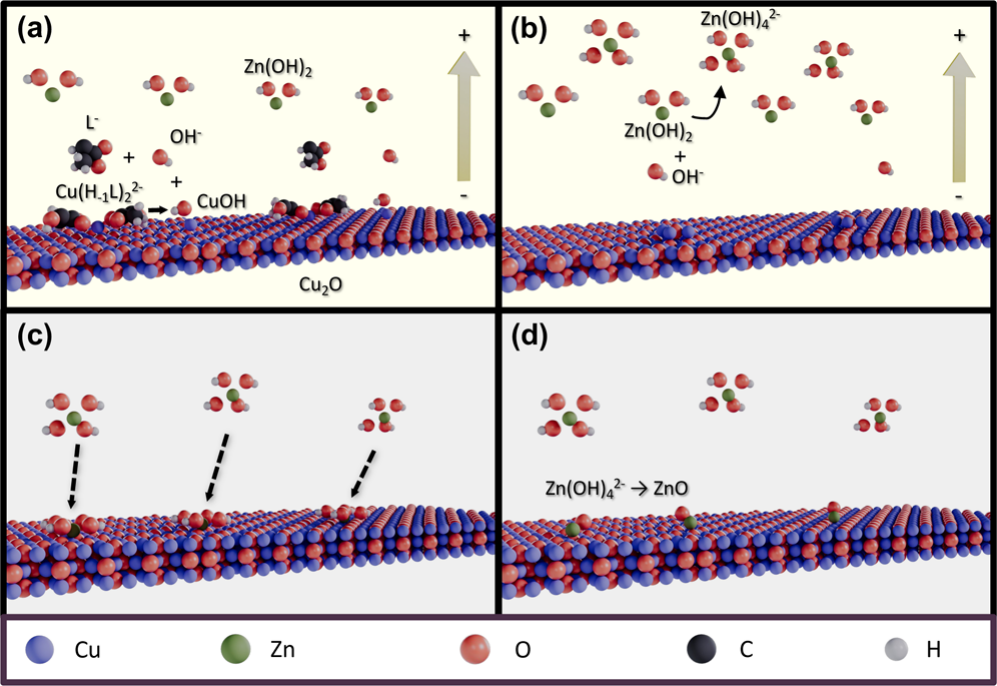
Schematic illustration of ZnO NPs forming on the surface of ZCO films. (a) Generation of excess OH^–^ ions and (b) formation of Zn­(OH)_4_^2–^ complexes during the growth of Cu_2_O; (c) adsorption of Zn­(OH)_4_^2–^ complexes on the Cu_2_O surface and (d) dehydration of Zn­(OH)_4_^2–^ into ZnO NPs.

Adding small amounts of ZnSO_4_ to the electrolyte did not alter the growth orientation and morphology of Cu_2_O crystallites. However, how ZnO NPs form on the ZCO film surface deserves further investigation. Figure 4 illustrates the tentative formation mechanism of ZnO NPs on the surface of ZCO films. It is worth noting that the production of ZnO NPs does not involve any charge transfer process. In a basic aqueous solution, Zn^2+^ ions react readily with hydroxyl ions (OH^–^) by forming Zn­(OH)_2_ precipitates in the solution (Figure 4a). However, Zn­(OH)_2_ will react with excess OH^–^ ions to form stable Zn­(OH)_4_^2–^ complexes in a high-pH solution.^41^ During the electrodeposition of Cu_2_O, the concentration of OH^–^ ions near the working electrode increases according to the eq 1 reaction, facilitating the formation of stable Zn­(OH)_4_^2–^ complexes in the electrolyte.^42^ These negatively charged Zn­(II)-complexes will be repelled from the surface of the working electrode during the growth of Cu_2_O (Figure 4b). After switching off the supplied electric current (Figure 4c,d), some Zn­(OH)_4_^2–^ ions adsorb onto the Cu_2_O surface and turn into ZnO following a spontaneous dehydration reaction below^43^

3 $$Zn{\left(OH\right)}_{4}^{2-}\to {ZnO}_{\left(s\right)}+H_{2}O+2OH^{-}$$

The ICP–MS analysis indicates that the Zn/Cu elemental ratios of the electrodeposited Cu_2_O and ZCO films increase proportionally with the concentration of Zn^2+^ in the electrolyte (Figure S3). The results suggest that the increasing Zn^2+^ concentration in the CuSO_4_ electrolyte promotes the generation of Zn­(OH)_4_^2–^ complexes, leading to a greater population of ZnO NPs on the Cu_2_O surface.

### 3.2 Photocatalytic and Electrochemical Properties of ZCO Films

Figure 5a shows the photocurrent–potential characteristics of the pristine Cu_2_O, ZCO-1 mM, ZCO-2 mM, and ZCO-3 mM samples in 1 M Na_2_SO_4_ solution under chopped illumination. Among the four samples, the peak photocurrent density measured at 0 V vs RHE follows the order of ZCO-2 mM > ZCO-1 mM > ZCO-3 mM > pristine Cu_2_O. The ZCO-2 mM sample exhibits the highest photocurrent density of 2.89 mA/cm^2^, which is a 40% improvement compared to the pristine Cu_2_O. A power-saved efficiency ϕ_saved_ = (V_saved_ *I*_p_)/*P*_t_ is commonly used to evaluate the efficiency of photocathodes, where V_saved_ is the voltage difference between the dark and light conditions under the same photocurrent *I*_p_, and *P*_t_ is the incident light power density. This metric quantifies how photoexcitation reduces the energy required to drive the electrochemical reaction.^44^ Figure 5b shows the plot of ϕ_saved_ against the applied potential for the pristine Cu_2_O, ZCO-1 mM, ZCO-2 mM, and ZCO-3 mM samples. The ZCO-2 mM enhances the power-saved efficiency to 3.5% from 2.5% for pristine Cu_2_O. The ZCO-3 mM sample does not provide any performance improvement and causes visible precipitation of Zn­(OH)_2_ in the bath. Therefore, the ZCO-2 mM sample represents the optimal balance between PEC performance and solution stability. The PEC stability of the Cu_2_O, ZCO-1 mM, and ZCO-2 mM samples was evaluated using the chronoamperometry technique. The samples were measured at 0 V vs RHE under chopped AM 1.5G illumination (Figure 5c). There is no significant difference in PEC stability between the pristine Cu_2_O and ZCO samples. Table 1 lists the photocurrent densities of various Cu_2_O/ZnO-based photocathodes reported in previous studies and this work. The synthesized ZCO films indeed exhibit better PEC properties than most other Cu_2_O/ZnO photocathodes. Most important of all, the ZCO films can be readily produced in a single electrodeposition cell without performing multiple deposition processes.

**Figure 5:**
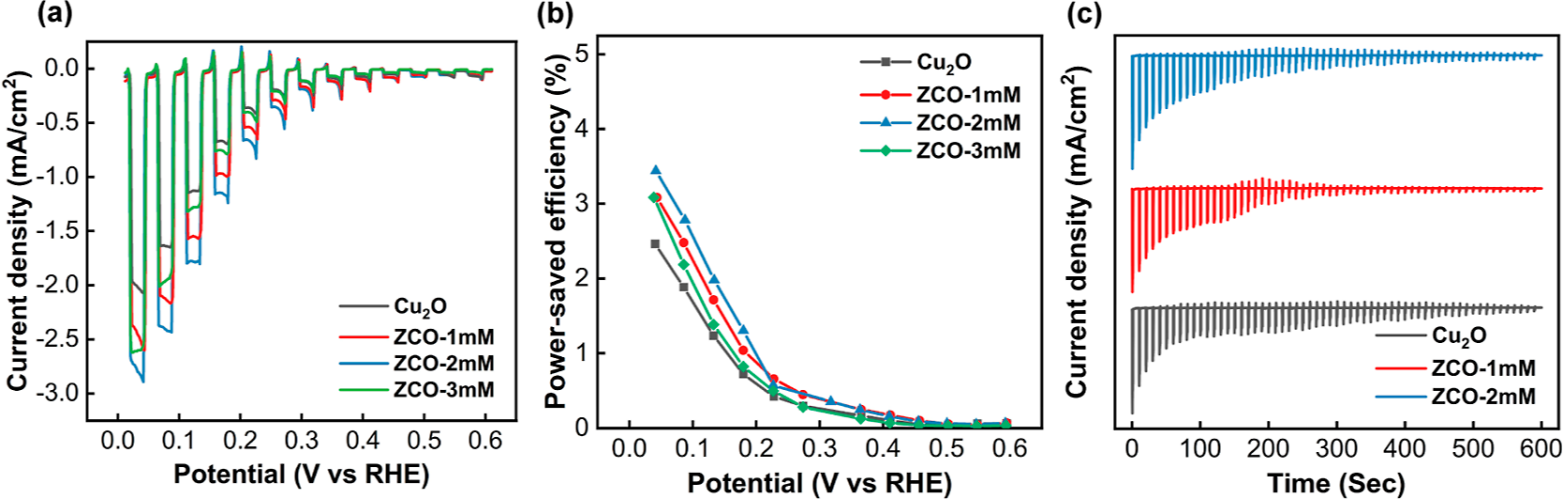
(a) LSV measurements of the Cu_2_O and ZCO samples in a 1 M Na_2_SO_4_ solution under chopped illumination; (b) A plot of the power-saved efficiency against the applied potential for the Cu_2_O and ZCO samples; (c) Chronoamperometry analysis of the Cu_2_O and ZCO samples measured at 0 V vs RHE under chopped AM 1.5G illumination.

**Table 1:** Maximum Photocurrent Densities of Various Cu_2_O/ZnO-Based Photoelectrodes Measured at 0 V Vs RHE in 1 M Na_2_SO_4_ Solution

photocathode	fabrication	*J* (mA/cm^2^)	ref
ITO/Ag/Cu_2_O/AZO	ALD	–0.4	^45^
ITO/Cu_2_O/ZnO	ALD	–1.6	^46^
ITO/Ag/Cu_2_O/AZO	RF sputtering	–0.25	^47^
Cu_2_O NW/ZnO NP	chemical bath	–3.4	^31^
Cu_2_O/ZnO/TiO_2_/Pt NWs	chemical bath	–2.8	^48^
FTO/Au/Cu_2_O/ZnO NP	one-step electrodeposition	–2.9	this work

An EIS analysis was conducted to provide insights into the electrochemical reaction kinetics of the ZnO NP-decorated Cu_2_O films. Figure 6 shows the frequency-dependent impedance characteristics of the pristine Cu_2_O, ZCO-1 mM, and ZCO-2 mM samples under dark and light conditions, represented in the form of Nyquist plots. The impedance data were fitted based on an equivalent Randles circuit model depicted in the inset, where the *R*_s_, *R*_ct_, CPE_dl_, and *W*_s_ are electrolyte resistance, charge transfer resistance to the electrolyte, constant phase element, and Warburg impedance, respectively.^34^ Table 2 lists the extracted components of all three samples. The diameter of the semicircles in the Nyquist plot can visualize the magnitude of *R*_ct_, which follows the order of Cu_2_O > ZCO-1 mM > ZCO-2 mM in both light and dark environments. The results indicate that the ZCO films have low charge transfer resistance at the film surface. This observation may be attributed to the modified band structure at the Cu_2_O/ZnO NPs interface. The type-II heterojunction between ZnO and Cu_2_O enables spatial separation of charge carriers, with photoelectrons migrating to the ZnO side and hole carriers to the Cu_2_O side.^29^ The bent band structure near the junction also creates a built-in electric field that facilitates the separation of photocarriers. This derivation is well supported by the reduced *R*_ct_ observed in the EIS measurements. This ZnO NPs modified Cu_2_O band structure will be discussed in the following section.

**Figure 6:**
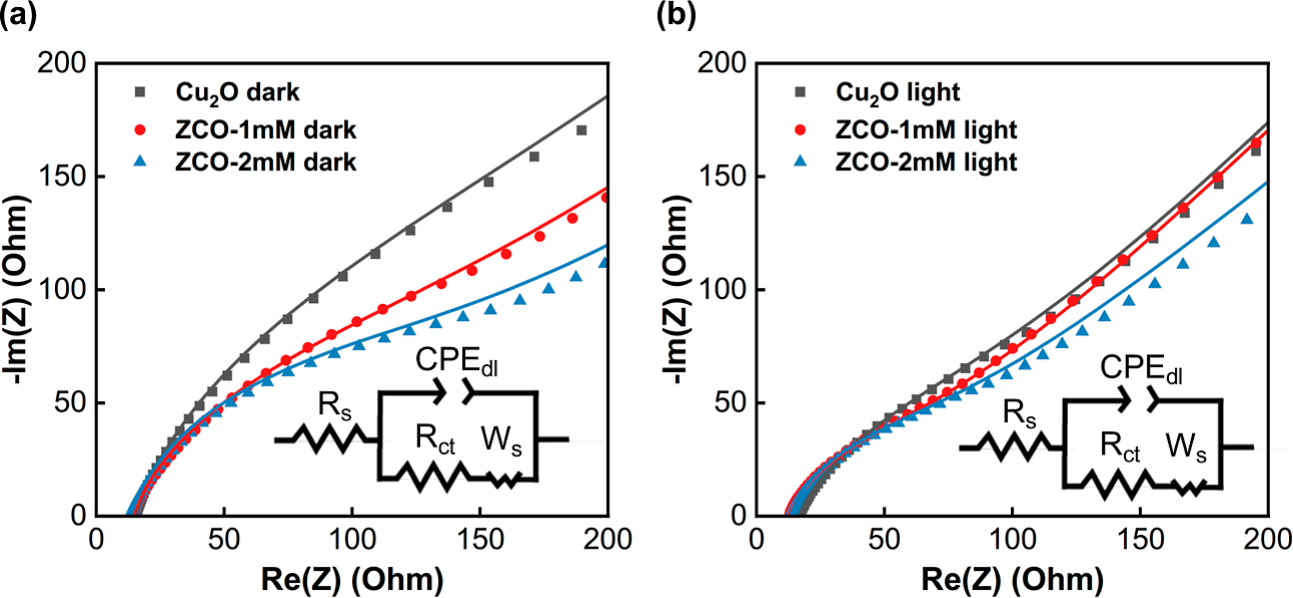
Nyquist plots of the pristine Cu_2_O, ZCO-1 mM, and ZCO-2 mM samples under (a) dark and (b) light conditions. The data were fitted using an equivalent Randles circuit model.

**Table 2:** Extracted Equivalent Randles Circuit Components for An Electrochemical System with Cu_2_O and ZCO Photocathodes in 1 M Na_2_SO_4_ Solution

	dark			light		
sample	*R*_s_ (Ω)	*R*_ct_ (Ω)	CPE_dl_ (μF/cm^2^)	*R*_s_ (Ω)	*R*_ct_ (Ω)	CPE_dl_ (μF/cm^2^)
Cu_2_O	15.4	217	11.3	16.6	115	32.3
ZCO-1 mM	14.3	140	16.8	12.5	77	33.9
ZCO-2 mM	13.6	117	10.0	13.2	67	20.3

The UV–visible absorption measurements were conducted for the pristine Cu_2_O, ZCO-1 mM, and ZCO-2 mM samples to reveal their electronic band structure information. All three samples show a similar optical bandgap value around 2.5 eV, as revealed in the Tauc plots (Figure 7a). The values agree reasonably with the reported bandgap for typical *p*-type Cu_2_O semiconductors (2–2.5 eV).^49^ The surface-distributed ZnO NPs basically do not change the bulk property of the Cu_2_O films. Consequently, a Mott–Schottky analysis was conducted to determine the flatband potentials (*E*_fb_) of all three samples in contact with the Na_2_SO_4_ solution (Figure 7b). The ZCO sample, particularly the ZCO-2 mM, shows a slightly higher flatband potential (*E*_fb_) than the pristine Cu_2_O sample. The pristine Cu_2_O films prepared in an alkaline solution are usually *p*-type semiconductors. When they encounter the Na_2_SO_4_ solution, charge transfer from the electrolyte to Cu_2_O causes the depletion of hole carriers and the downward band bending at the Cu_2_O/electrolyte interface. In the *p*-Cu_2_O/*n*-ZnO NPs/electrolyte system, hole carriers near the Cu_2_O/ZnO interface are more extensively depleted, causing further band bending and a higher *E*_fb_ for the ZCO-2 mM sample (Figure 7c). Since the carrier concentration (*N*_A_) can be extracted from the slope of the Mott–Schottky plot, the Fermi energies of the pristine Cu_2_O and ZCO films were calculated using the following equation.

4 $$E_{F}=E_{V}+k_{B}T\ln\left(\frac{N_{V}}{N_{A}}\right),,where\;N_{V}=2{\left(\frac{2\pi m^{*}k_{B}T}{h^{2}}\right)}^{3/2}$$

**Figure 7:**
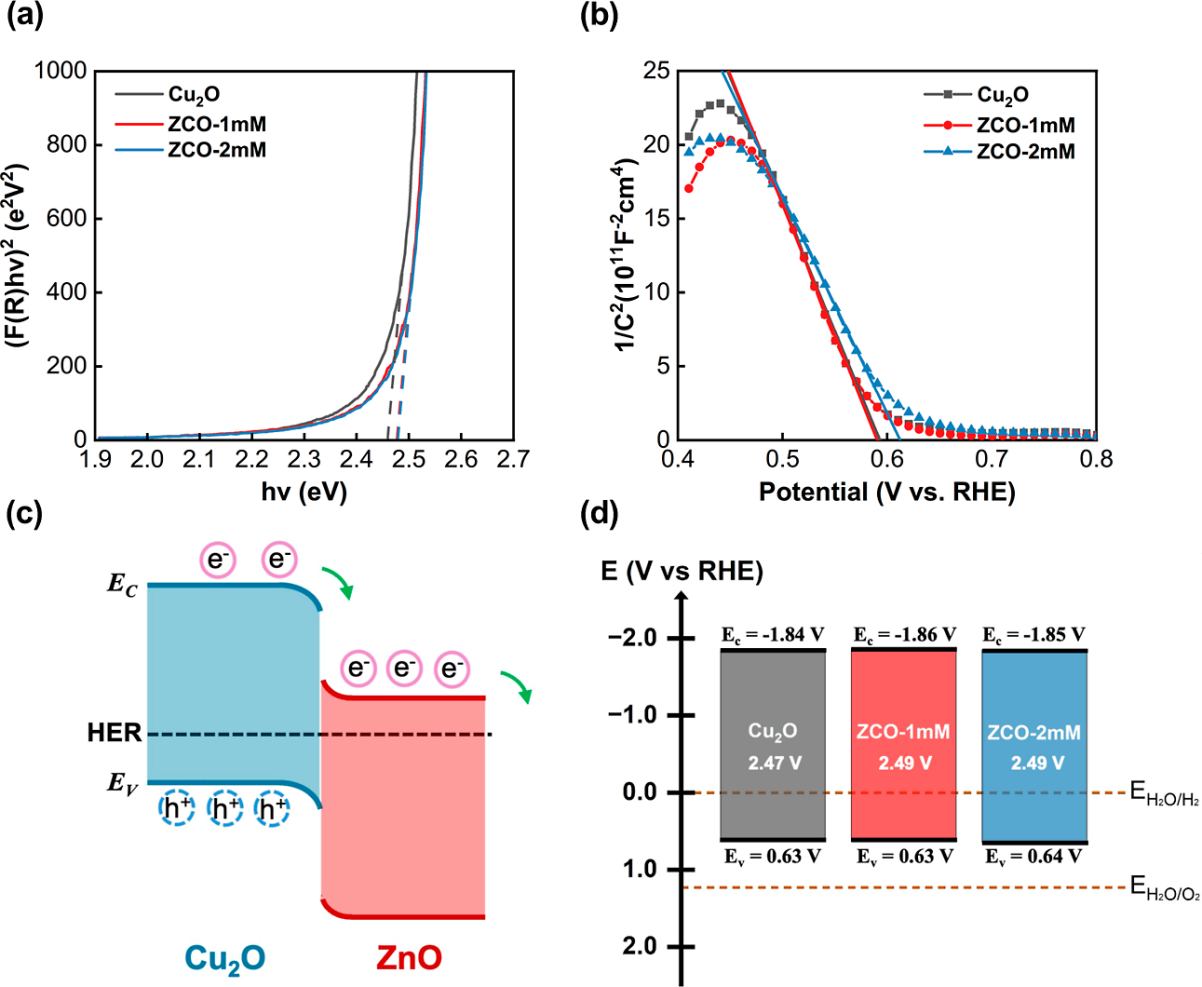
(a) Tauc plot, (b) Mott–Schottky analysis, (c) Schematic band diagrams, and (d) Relative electronic band positions of the Cu_2_O, ZCO-1 mM, and ZCO-2 mM samples.

The band structures of the pristine Cu_2_O, ZCO-1 mM, and ZCO-2 mM samples were thus determined based on the measured bandgap and the Fermi energy (Figure 7d). The built-in electric field at the Cu_2_O/ZnO interfaces facilitates the photoelectrons to flow from Cu_2_O to the electrolyte, thereby enhancing the separation efficiency of photocarriers in the Cu_2_O.

The recombination dynamics of photocarriers in the Cu_2_O and ZCO films after excitation were investigated using photoluminescence (PL) spectroscopy. Figure 8a shows the PL spectra of the pristine Cu_2_O, ZCO-1 mM, and ZCO-2 mM samples measured at room temperature. One emission band at the wavelength of 628 nm corresponds to the free exciton emission in Cu_2_O, while the other band at the wavelength around 700 nm–900 nm is associated with the bound exciton emission around oxygen vacancy defects in Cu_2_O.^50^ It has been reported that the exciton emissions at 750 and 850 nm may involve the double-charged oxygen vacancies (V_O_^2+^) and the single-charged oxygen vacancies (V_O_^+^), respectively.^51^ Based on the defect-mediated emission spectra, V_O_^+^ is expected to be located at a deeper energy level than V_O_^2+^. Note that the V_O_^+^/V_O_^2+^-mediated emission band shows a slight red shift with the modification of ZnO NPs on the Cu_2_O surface, particularly for the ZCO-2 mM sample. It implies that the relative population of bound excitons around V_O_^+^ may become more predominant in the ZCO samples (Figure S4). This result suggests that some photoelectrons proceed with a nonradiative transition from V_O_^2+^ state to the V_O_^+^ state before the recombination with hole carriers. The shift in recombination dynamics of photocarriers was also investigated using TRPL spectroscopy. Figure 8b shows the TRPL decay traces of the pristine Cu_2_O, ZCO-1 mM, and ZCO-2 mM samples measured at room temperature. The time constants and amplitudes were extracted by fitting the TRPL data using a biexponential equation. Table 3 lists the extracted time constants and amplitudes of various Cu_2_O-based samples. The fast photoluminescence process (A_1_ and τ_1_) is likely associated with the direct band-to-band recombination process in Cu_2_O, while the slow process (*A*_2_ and τ_2_) may indicate the oxygen vacancy defect-mediated emission process in Cu_2_O.^52^ The average carrier lifetime (τ_ave_) for the bimodal photocarrier recombination process is formulated by^53^

5 $$\tau_{ave}=\left(A_{1}\tau_{1}^{2}+A_{2}\tau_{2}^{2}\right)/\left(A_{1}\tau_{1}+A_{2}\tau_{2}\right)$$

**Figure 8:**
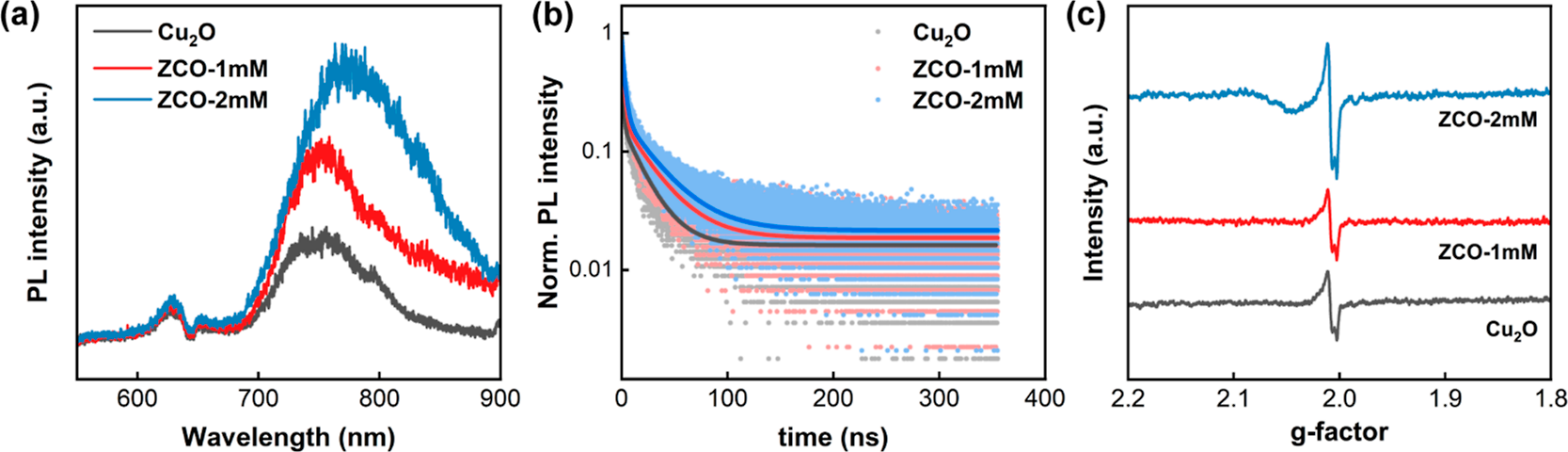
(a) PL spectra, (b) TRPL spectra, and (c) electron paramagnetic resonance spectra of the pristine Cu_2_O, ZCO-1 mM, and ZCO-2 mM samples.

**Table 3:** Time Constants and Amplitudes Extracted From the TRPL Decay Traces of the Pristine Cu_2_O, ZCO-1 mM, and ZCO-2 mM Samples

sample	*A*_1_	τ_1_ (ns)	*A*_2_	τ_2_ (ns)	τ_ave_
Cu_2_O	0.83	1.12	0.17	19.72	15.61
ZCO-1 mM	0.80	2.22	0.20	27.67	21.45
ZCO-2 mM	0.83	2.69	0.17	31.05	24.18

The τ_ave_ of photocarriers in Cu_2_O-based films apparently increases with the modification of ZnO NPs. It has been proposed that dispersed oxygen vacancy defects will capture photoelectrons, and these trapped electrons must travel over long distances through multiple hopping and tunneling events to combine with holes, leading to a prolonged carrier lifetime.^54^ The increased defect-mediated emission spectral intensity (Figure 8a) and the prolonged carrier lifetime (Figure 8b) suggest that the concentration of oxygen vacancies will likely increase with the introduction of ZnO NPs in the Cu_2_O-based films. When an unpaired electron is captured by an oxygen vacancy, it generates a magnetic moment that resonates at a specific microwave frequency under an external magnetic field. The EPR spectra offer detailed insights into the interactions between electron carriers and crystal defects in solids. Figure 8c shows the variation of EPR spectral intensity against the *g*-factor for the Cu_2_O and ZCO films studied. The *g*-factor is a parameter that connects an electron’s magnetic moment to its angular momentum. The *g*-factor for an unpaired electron trapped in an oxygen vacancy defect in Cu_2_O is typically close to the free-electron value of 2.0023.^55^ The pristine Cu_2_O and ZCO films indeed exhibit strong resonant signals with a *g*-factor of 2.009. This EPR spectral intensity can be linked to the concentration of oxygen vacancies present in Cu_2_O. Among the three samples, the ZCO-2 mM shows the strongest resonant signal, indicating it has the highest concentration of oxygen vacancy defects in Cu_2_O.

Now, the remaining question to be answered would be how the ZnO NPs affect the oxygen vacancy defects in Cu_2_O for the ZCO films. As mentioned previously, Cu­(II)-lactate complexes convert into Cu­(OH) on the Cu_2_O surface, accompanied by the creation of OH^–^ ions. The pH value in the electrolyte near the working electrode thus increases with the growth of Cu_2_O. However, by adding ZnSO_4_ to the electrolyte, Zn^2+^ ions can combine with excess OH^–^ ions to form Zn­(OH)_4_^2–^ complexes, leading to an OH^–^ ion-deficient environment near the working electrode. The formation of oxygen vacancies in electrodeposited Cu_2_O is highly sensitive to the pH value of the electrolyte. A theoretical study shows that low pH environments have decreased formation energy for oxygen vacancies in Cu_2_O.^56^ Therefore, the OH^–^ ion-deficient environment will favor the formation of oxygen vacancy defects in Cu_2_O due to the existence of Zn­(OH)_4_^2–^ complexes. This explains why the ZCO-2 mM sample displays the highest oxygen vacancy concentration in Cu_2_O. These oxygen vacancies in Cu_2_O serve as electron trapping centers, preventing the rapid recombination of photocarriers. Thus, the photoelectrons in the ZCO films exhibit longer carrier lifetimes and a higher probability of reducing water molecules to hydrogen.

## 4 Conclusions

A facile one-step electrodeposition process is employed to prepare the Zn NPs decorated Cu_2_O photocathodes. The ZnO NPs primarily reside on the surface of Cu_2_O due to the stable Zn­(OH)_4_^2–^ complexes being repelled from the working electrode during the electrodeposition of Cu_2_O. The ZnO NPs form when Zn­(OH)_4_^2–^ complexes adsorb on the Cu_2_O surface and undergo the spontaneous dehydration reaction after switching off the electrode potential. The ZnO NP-decorated Cu_2_O films have the preferred type II heterojunction to enhance the separation of photocarriers in the illuminated Cu_2_O. The increased oxygen vacancy defects also help enhance the photocatalytic property of ZCO films by raising the photocarrier lifetime. The synthesized ZCO films exhibit a 40% increase in photocurrent density compared to the pristine Cu_2_O photocathode.

## Supplementary Material



## Notes

Log–scale GIXRD pattern; O 1s XPS depth profiling analysis; ICP–MS analysis; deconvolution of PL spectra (PDF)
