# Supplementary material for: Single-Step Electrodeposition of ZnO Nanoparticles Decorated (111)-Textured Cu2O Films with Enhanced Photoelectrochemical Properties
Source: Inorg Chem. 2025 Aug 12;64(33):16950–9. doi: 10.1021/acs.inorgchem.5c02573 (PMC12381859; doi:10.1021/acs.inorgchem.5c02573)
Supplement: Supplementary file 1 [file ic5c02573_si_001.pdf]

SUPPORTING INFORMATION

Single-Step Electrodeposition of ZnO Nanoparticles Decorated (111)-Textured Cu_2O Films with Enhanced Photoelectrochemical Properties

*Yu-Hao Huang[1], Yung-Tang Chuang[2], Hao-Wu Lin[2], and Chien-Neng Liao[*1,2]*

[1] College of Semiconductor Research, National Tsing Hua University, Hsinchu 30013, Taiwan

[2] Department of Materials Science and Engineering, National Tsing Hua University, Hsinchu 30013, Taiwan

Corresponding Author

*E-mail: cnliao@mx.nthu.edu.tw

Supplementary Figures

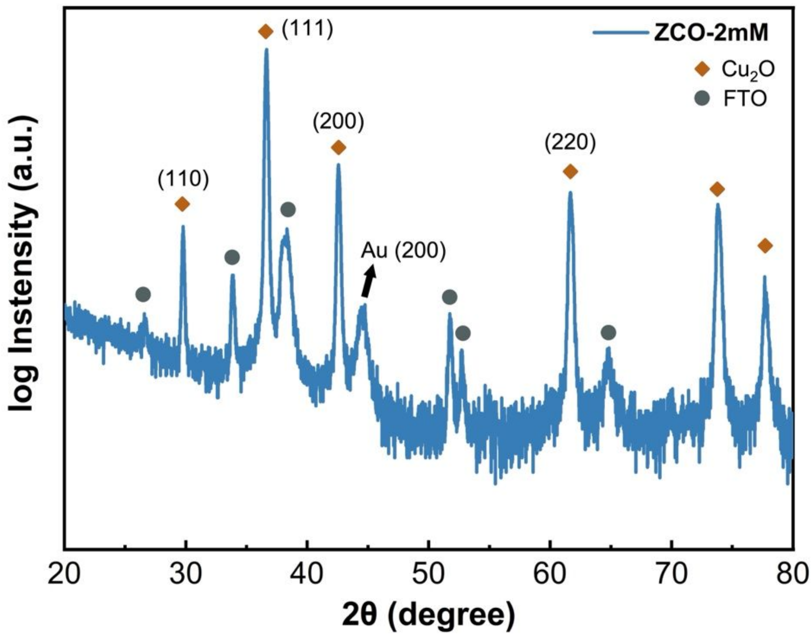

Figure S1. Log–scale GIXRD pattern for the ZCO-2mM sample

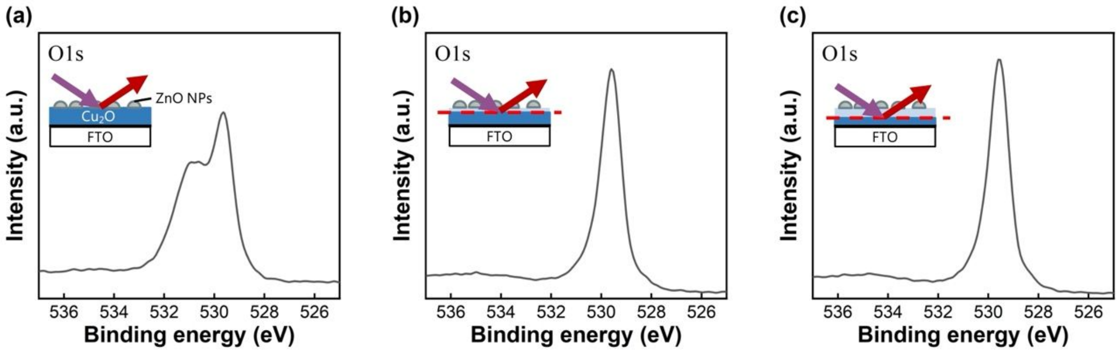

Figure S2. An O1s XPS depth profiling analysis of the ZCO-2mM sample subjected to Ar ion milling for (a) 0 min, (e) 3 min, and (f) 5 min.

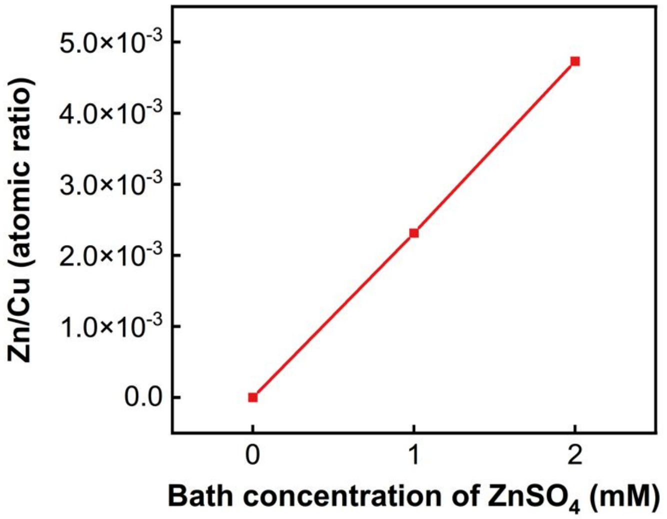

Figure S3. ICP-MS analysis for the Cu_2O-based films fabricated using the $CuSO_4$ electrolytes with different bath concentrations of $ZnSO_4$

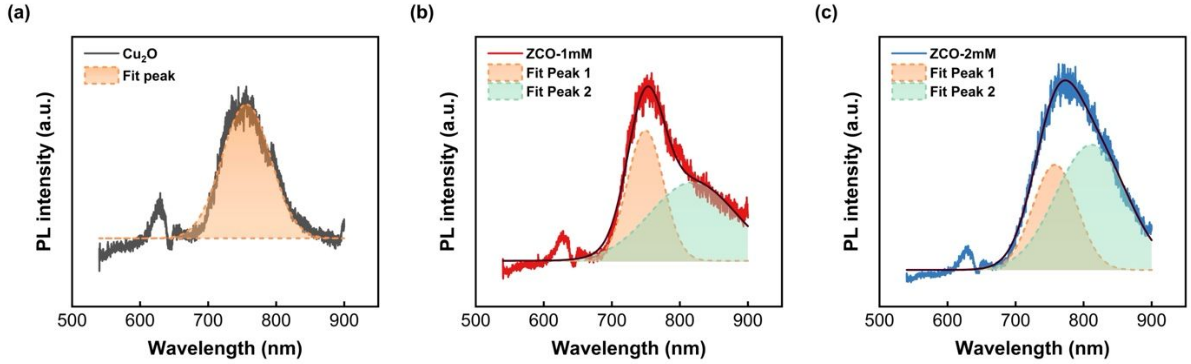

Figure S4. Deconvolution of PL spectra of the Cu_2O, ZCO-1mM, and ZCO-2mM samples.
